# Learning and stabilization of winner-take-all dynamics through interacting excitatory and inhibitory plasticity

**DOI:** 10.3389/fncom.2014.00068

**Published:** 2014-07-08

**Authors:** Jonathan Binas, Ueli Rutishauser, Giacomo Indiveri, Michael Pfeiffer

**Affiliations:** ^1^Institute of Neuroinformatics, University of Zurich and ETH ZurichZurich, Switzerland; ^2^Department of Neurosurgery and Department of Neurology, Cedars-Sinai Medical CenterLos Angeles, CA, USA; ^3^Computation and Neural Systems Program, Division of Biology and Biological Engineering, California Institute of TechnologyPasadena, CA, USA

**Keywords:** winner-take-all, competition, plasticity, self-organization, contraction theory, canonical microcircuits, inhibitory plasticity

## Abstract

Winner-Take-All (WTA) networks are recurrently connected populations of excitatory and inhibitory neurons that represent promising candidate microcircuits for implementing cortical computation. WTAs can perform powerful computations, ranging from signal-restoration to state-dependent processing. However, such networks require fine-tuned connectivity parameters to keep the network dynamics within stable operating regimes. In this article, we show how such stability can emerge autonomously through an interaction of biologically plausible plasticity mechanisms that operate simultaneously on all excitatory and inhibitory synapses of the network. A weight-dependent plasticity rule is derived from the triplet spike-timing dependent plasticity model, and its stabilization properties in the mean-field case are analyzed using contraction theory. Our main result provides simple constraints on the plasticity rule parameters, rather than on the weights themselves, which guarantee stable WTA behavior. The plastic network we present is able to adapt to changing input conditions, and to dynamically adjust its gain, therefore exhibiting self-stabilization mechanisms that are crucial for maintaining stable operation in large networks of interconnected subunits. We show how distributed neural assemblies can adjust their parameters for stable WTA function autonomously while respecting anatomical constraints on neural wiring.

## 1. Introduction

Competition through shared inhibition is a powerful model of neural computation (Maass, 2000; Douglas and Martin, 2007). Competitive networks are typically composed of populations of excitatory neurons driving a common set of inhibitory neurons, which in turn provide global negative feedback to the excitatory neurons (Amari and Arbib, 1977; Douglas and Martin, 1991; Hertz et al., 1991; Coultrip et al., 1992; Douglas et al., 1995; Hahnloser et al., 2000; Maass, 2000; Rabinovich et al., 2000; Yuille and Geiger, 2003; Rutishauser et al., 2011). Winner-take-all (WTA) networks are one instance of this circuit motif, which has been studied extensively. Neurophysiological and anatomical studies have shown that WTA circuits model essential features of cortical networks (Douglas et al., 1989; Mountcastle, 1997; Binzegger et al., 2004; Douglas and Martin, 2004; Carandini and Heeger, 2012). An individual WTA circuit can implement a variety of non-linear operations such as signal restoration, amplification, filtering, or max-like winner selection, e.g., for decision making (Hahnloser et al., 1999; Maass, 2000; Yuille and Geiger, 2003; Douglas and Martin, 2007). The circuit plays an essential role in both early and recent models of unsupervised learning, such as receptive field development (von der Malsburg, 1973; Fukushima, 1980; Ben-Yishai et al., 1995), or map formation (Willshaw and Von Der Malsburg, 1976; Amari, 1980; Kohonen, 1982; Song and Abbott, 2001). Multiple WTA instances can be combined to implement more powerful computations that cannot be achieved with a single instance, such as state dependent processing (Rutishauser and Douglas, 2009; Neftci et al., 2013). This modularity has given rise to the idea of WTA circuits representing *canonical microcircuits*, which are repeated many times throughout cortex and are modified slightly and combined in different ways to implement different functions (Douglas and Martin, 1991, 2004; Rutishauser et al., 2011).

In most models of WTA circuits the network connectivity is given a priori. In turn, little is known about whether and how such connectivity could emerge without precise pre-specification. In this article we derive analytical constraints under which local synaptic plasticity on all connections of the network tunes the weights for WTA-type behavior. This is challenging as high-gain WTA operation on the one hand, and stable network dynamics on the other hand, impose diverging constraints on the connection strengths (Rutishauser et al., 2011), which should not be violated by the plasticity mechanism. Previous models like Jug et al. (2012) or Bauer (2013) have shown empirically that functional WTA-like behavior can arise from an interplay of plasticity on excitatory synapses and homeostatic mechanisms. Here, we provide a mathematical explanation for this phenomenon, using a mean-field based analysis, and derive conditions under which biologically plausible plasticity rules applied to all connections of a network of randomly connected inhibitory and excitatory units produce a functional WTA network with structured connectivity. Due to plastic inhibitory synapses, convergence of the model does not rely on constant, pre-defined inhibitory weights or other common assumptions for WTA models. We prove that the resulting WTA circuits obey stability conditions imposed by contraction analysis (Lohmiller and Slotine, 1998; Rutishauser et al., 2011). This has important implications for the stability of larger networks composed of multiple interconnected WTA circuits, and thus sheds light onto the mechanisms responsible for the emergence of both local functional cortical microcircuits and larger distributed coupled WTA networks.

This article is structured as follows: We first define the network and plasticity models in sections 2.1 to 2.3. Our main analytical results are given in sections 2.4 and 2.5, and illustrated with simulation results in section 2.6. The results are discussed in section 3, and detailed derivations of the analytical results can be found in section 4.

## 2. Results

### 2.1. Network topology

**Figure 1:**
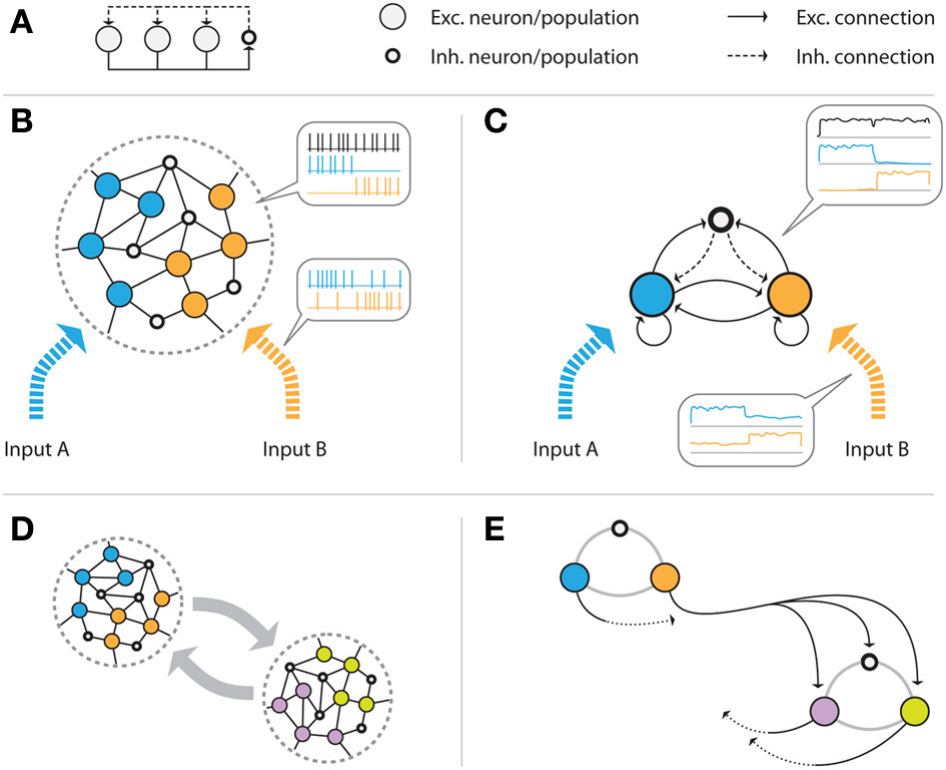
**Illustration of the network model. (A)** Abstract representation of a WTA circuit, where several excitatory units project onto a common inhibitory unit, and receive global inhibitory feedback from that unit. **(B)** Example volume of the generic cortical structure that is assumed, consisting of (initially randomly connected) excitatory (pyramidal) cells and inhibitory interneurons. The color of the cells indicates the input channel they are connected to: some cells only receive input from the blue, others from the orange source. It is assumed that the volume is sufficiently small, such that all excitatory cells can be reached by the (short-ranged) axons of the inhibitory cells. If the connection strengths are tuned appropriately, the population receiving the stronger input signal will suppress the response of the weaker population via the global inhibitory feedback. **(C)** shows the mean field model of the same network that we construct by grouping excitatory neurons by their input source. The three resulting excitatory and interneuron populations are connected in an all-to-all fashion. **(D,E)** show multiple, distant volumes which are connected via long-range excitatory connections. Projections from one volume to another connect to all cells of the target volume. In **(E)**, the two subgroups are approximated by networks of the type shown in **(C)**, consisting of one inhibitory and several excitatory populations. The black, solid arrows represent exemplary excitatory connections from one population of one group to all populations of the other group. Equivalent connections, indicated by dotted arrows, exist for all of the excitatory populations.

In its simplest abstract form, a WTA circuit (Figure 1A) consists of a number of excitatory units that project onto a common inhibitory unit. This unit, in turn, provides recurrent inhibitory feedback to all excitatory units. Given appropriate connection strengths, such inhibition makes the excitatory units compete for activation in the sense that the unit receiving the strongest input signal will suppress the activation of all other units through the inhibitory feedback loop, and “win” the competition.

We design a biologically plausible network by taking into account that inhibitory feedback is local, i.e., it only affects cells within a cortical volume that is small enough such that the relatively short inhibitory axonal arbors can reach their targets. We assume excitatory and inhibitory neurons in this volume to be connected randomly (see Figure 1B). Furthermore, we assume that there are a finite number of different input signals, each activating a subset of the excitatory cells in the volume. We construct a mean-field model by grouping the excitatory neurons for each driving input stimulus, summarizing the activity of each group of cells by their average firing rate. This results in a simplified population model of the network which—in the case of two different input signals—consists of two excitatory populations (one for each input), and one inhibitory population (see Figure 1C). We assume full recurrent connectivity between all populations. This scheme can easily be extended toward more input groups. In particular, if an excitatory group receives multiple inputs, it can be modeled as a new class.

Since inhibitory axons are (typically) short-range, distant populations can communicate only via excitatory projections. We combine multiple local circuits of the form shown in Figures 1B,C by introducing excitatory long-range connections between them, as illustrated in Figure 1D. Specifically, we add projections from the excitatory populations of one local group to all excitatory and inhibitory populations of the other group. A similar connectivity scheme for implementing distributed WTA networks has been proposed by Rutishauser et al. (2012). Unlike their model, our network does not require specific wiring, but rather targets any potential cell in the other volume. We will show in section 2.4.4 that this is sufficient to achieve competition between units of spatially distributed WTA circuits.

### 2.2. Network dynamics

The activation of a neural populations *x*_*i*_, which can be excitatory or inhibitory, is described by

(1) $$\tau_{i}{\dot{x}}_{i}(t)=-x_{i}(t)+{\left[\sum_{j}w_{ij}x_{j}(t)+I_{\text{ext},i}(t)-T_{i}\right]}_{+},$$

where τ_*i*_ is the time constant of the population, *w*_*ij*_ is the weight of the incoming connection from the *j*th population, *I*_ext,*i*_(*t*) is an external input given to the population, and *T*_*i*_ is the activation threshold. Furthermore, [*v*]_+_ := max (0, *v*) is a half-wave rectification function, preventing the firing rates from taking negative values. Assuming identical time constants for all populations, i.e., τ_*i*_ = τ for all *i*, the dynamics of the full system can be written as

(2) $$\tau \dot{x}(t)=-x(t)+{\left[Wx(t)+I_{\text{ext}}(t)-T\right]}_{+},$$

where ***x*** = (*x*_1_, …, *x*_*N*_) are the firing rates of the respective populations (excitatory and inhibitory), ***W*** is the connectivity matrix (describing local excitatory, local inhibitory, and long-range excitatory connections), ***I***_ext_(*t*) is a vector of external inputs, and ***T*** = (*T*_1_, …, *T*_*N*_) are the activation thresholds of the populations. For the single local microcircuit shown in Figure 1C, for example, ***W*** would be a 3-by-3 matrix with all entries *w*_*ij*_ non-zero except for the inhibitory to inhibitory coupling. For two coupled microcircuits as in Figure 1E, the connectivity matrix consists of 4 blocks, with the diagonal blocks describing local connectivity, and the off-diagonal blocks describing long-range projections from excitatory units to the other circuit.

### 2.3. Plasticity mechanisms and weight dynamics

In our model, we assume that all connections *w*_*ij*_ in Equation (2) are plastic, and are subject to the following weight update rule:

(3) $$\dot{w}=\tau_{\text{s}}^{2}x_{\text{pre}}x_{\text{post}}\left(x_{\text{post}}(w_{\text{max}}-w)-(\Theta_{w}+A_{w}x_{\text{pre}})w\right).$$

**Figure 2:**
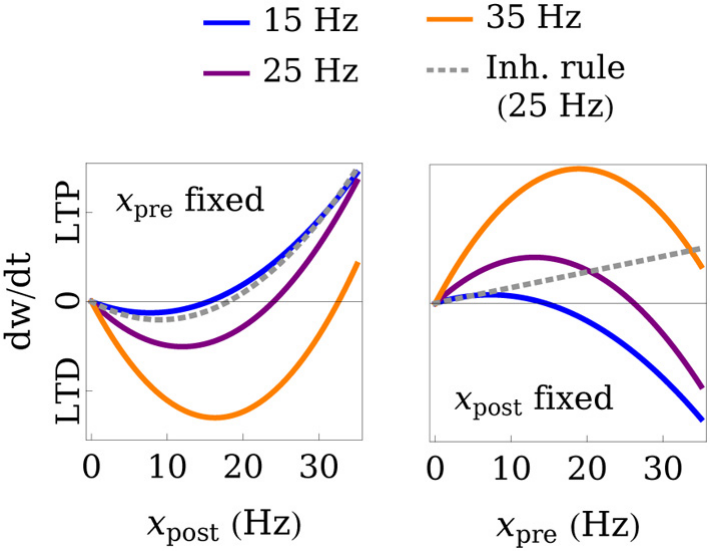
**Illustration of the learning rule**. The weight change *dw/dt* is plotted as a function of the post- and presynaptic firing rate for fixed pre- (left) or postsynaptic (right) rates. The gray, dashed line shows the rule that we use for inhibitory connections and whose threshold for LTP, in contrast to excitatory connections, does not depend on the presynaptic rate. The black line marks the transition between LTD and LTP. In this example, the parameters of the learning rules were set to Θ_exc_ = 6 Hz, Θ_inh_ = 18 Hz, *A*_exc_ = 2, *w*_max_ = 4, and the weight value was fixed at *w*_max_/3 for excitatory and *w*_max_/2 for inhibitory connections.

Here, *x*_pre_ and *x*_post_ are the pre- and postsynaptic firing rates, respectively, *w*_max_ is the maximum possible weight value, and Θ_*w*_, *A*_*w*_, and τ_*s*_ are positive constants, which we set to values that are compatible with experimental findings (see Table 1). The learning rate is determined by τ_*s*_, and Θ_*w*_ and *A*_*w*_ determine the point at which the rule switches between depression (LTD) and potentiation (LTP). We will show that in a plastic network, global stability and circuit function are determined exclusively by those plasticity parameters. The plasticity rule is derived from the mean-field approximation of the triplet STDP rule by Pfister and Gerstner (2006), which we augment with a weight-dependent term, effectively limiting the weight values to the interval [0, *w*_max_]. A more detailed derivation of the learning rule can be found in the Methods (section 4.1). The parameters Θ_*w*_ and *A*_*w*_ are set differently for excitatory and inhibitory connections, leading to two types of simultaneously active plasticity mechanisms and weight dynamics, even though the same learning equation is used. We set Θ_*w*_ = Θ_exc_ and *A*_*w*_ = *A*_exc_ for all excitatory connections, and Θ_*w*_ = Θ_inh_ and *A*_*w*_ = *A*_inh_ for all inhibitory connections. In particular, we assume *A*_inh_ to take very low values and set *A*_inh_ = 0 in our analysis, effectively eliminating any dependence of the fixed point of inhibitory weights on the presynaptic rate. According to fits of the parameters to experimental data (see Table 1), this is a plausible assumption. For the sake of simplicity, we also assume the maximum possible weight value *w*_max_ to be the same for all excitatory and inhibitory connections. Figure 2 illustrates the weight change as a function of the pre- and postsynaptic activity.

**Table 1:** **Learning rule parameters *A*^±^_2_, and *A*^±^_3_ from Pfister and Gerstner (2006)**.

**Model**	**A**^**+**^_**2**_	**A**^**+**^_**3**_	**A**^−^_**2**_	**A**^−^_**3**_	**Θ**	**A**	**τ**^**2**^_**s**_
All-to-all	5 × 10^−10^	6.2 × 10^−3^	7 × 10^−3^	2.3 × 10^−4^	18.19	0.06	1.3 × 10^−5^
Nearest spike	8.8 × 10^−11^	5.3 × 10^−2^	6.6 × 10^−3^	3.1 × 10^−3^	6.24	2.09	3.6 × 10^−5^

*The values of Θ, A, and τ_s_ for the plasticity rule Equation (3) have been computed using Equations (14) to (16). The data corresponds to fits of the triplet Spike-Timing Dependent Plasticity (STDP) model with all-to-all spike interactions (first row) and with nearest spike interactions (second row) to recordings from plasticity experiments in rat visual cortex. Note that the time constants τ_x,y_ and τ_±_ are not reproduced here. However, they all are of the order of hundreds of milliseconds and can be found in Pfister and Gerstner (2006). In our simulations, we use parameters very similar to the “all-to-all” parameters for inhibitory connections, while for excitatory connections we use ones that are close to the “nearest spike” parameters*.

### 2.4. Stability analysis

The WTA circuit is assumed to function correctly if it converges to a stable state that represents the outcome of the computation it is supposed to perform. Conditions under which these networks converge to their (single) attractor state exponentially fast were previously derived by Rutishauser et al. (2011). Here, we extend those results to plastic networks and express stability criteria in terms of global learning rule parameters, rather than individual weight values. We first describe criteria for the stabilization of the network and learning rule dynamics, then derive from them conditions on the learning rule parameters. Our analysis leads to very simple sufficient conditions that ensure the desired stable WTA behavior.

The dynamics of the network activation and the weights are given by Equations (2) and (3), respectively. In the following, we will denote them by ***f*** and ***g***, so the full dynamics can be written as a coupled dynamical system

(4) $$\dot{x}=f(x,w),$$

(5) $$\dot{w}=g(x,w),$$

where ***f*** corresponds to the right hand side of Equation (2), and ***g*** combines the update rules for all weights (with different sets of parameters for excitatory and inhibitory connections) in one vector-valued function. We first restrict our analysis to the simplest case of a single winning excitatory population and derive conditions under which the plastic network converges to its fixed point. Later, we extend our analysis to larger systems of multiple coupled excitatory populations.

#### 2.4.1. Analysis of single-node system

Let us first consider a simplified system, in which only one excitatory population is active, e.g., because one population receives much more external input than all others, and the inhibitory feedback suppresses the other populations. As silent populations neither contribute to the network dynamics nor to the weight dynamics, they can be excluded from the analysis. We can therefore reduce the description of the system to a single excitatory population *x*_E_, and an inhibitory population *x*_I_, together with the connections *w*_E→E_, *w*_E→I_, and *w*_I→E_ between them.

For a given set of (fixed) weights ***w***_*c*_, Rutishauser et al. (2011) have shown by means of contraction theory (Lohmiller and Slotine, 1998) that the system of network activations ***ẋ*** = ***f***(***x, w***_*c*_) converges to its fixed point x^*^ exponentially fast if its generalized Jacobian is negative definite. In our case, this condition reduces to

(6) $$\text{Re}\left(w_{\text{E}\to \text{E}}-2+{\left(w_{\text{E}\to \text{E}}^{2}-4w_{\text{I}\to \text{E}}w_{\text{E}\to \text{I}}\right)}^{1/2}\right)<0.$$

If condition (6) is met, the system is called contracting and is guaranteed to converge to its attractor state

(7) $$x_{\text{E}}^{*}=\Lambda I_{\text{ext}},$$

(8) $$x_{\text{I}}^{*}=\Lambda w_{\text{E}\to \text{I}}I_{\text{ext}},$$

exponentially fast for any constant input *I*_ext_, where the contraction rate is given by the left hand side of (6), divided by 2τ. Here, Λ = (1 − *w*_E→E_ + *w*_E→I_ *w*_I→E_)^−1^ corresponds to the network gain. A more detailed derivation of the fixed point can be found in section 4.2. Note that we have set the activation threshold *T* equal to zero and provide external input *I*_ext_ to the excitatory population only. This simplifies the analysis but does not affect our results qualitatively.

#### 2.4.2. Decoupling of network and weight dynamics

In the following, we assume that the population dynamics is contracting, i.e., that condition (6) is met, to show that the plasticity dynamics Equation (5) drives the weights ***w*** to a state that is consistent with this condition. Essentially, our analysis has to be self-consistent with respect to the contraction of the activation dynamics. If we assume ***f*** and ***g*** to operate on very different timescales, we can decouple the two systems given by Equations (4) and (5). This is a valid assumption since neural (population) dynamics vary on timescales of tens or hundreds of milliseconds (see **Figure 5** for typical timescales of our system), while synaptic plasticity typically acts on timescales of seconds or minutes. This means that from the point of view of the weight dynamics ***g*** the population activation is at its fixed point x^*^ almost all the time, because it converges to that point exponentially fast. We can thus model the activation dynamics as a quasi-static system, and approximate the learning dynamics as a function of the fixed point of the activation instead of the instantaneous activation.

(9) $$g(x,w)\approx g(x^{*},w),$$

The fixed point of this simplified system is found by setting ***g***(x^*^, ***w***) = **0**, and according to Equation (3) is given by

(10) $$w^{*}=\frac{w_{\text{max}}\;x_{\text{post}}^{*}}{\Theta_{w}+A_{w}\;x_{\text{pre}}^{*}+x_{\text{post}}^{*}}.$$

Combining this expression with Equations (7) and (8) leads to a system of non-linear equations that can be solved for the fixed point weights w^*^_E→E_, w^*^_E→I_, w^*^_I→E_, and activations x^*^_E_, and x^*^_I_. These values solely depend on the learning rule parameters Θ_*w*_, *A*_*w*_, *w*_max_, and the external (training) input *I*_ext_.

**Figure 3:**
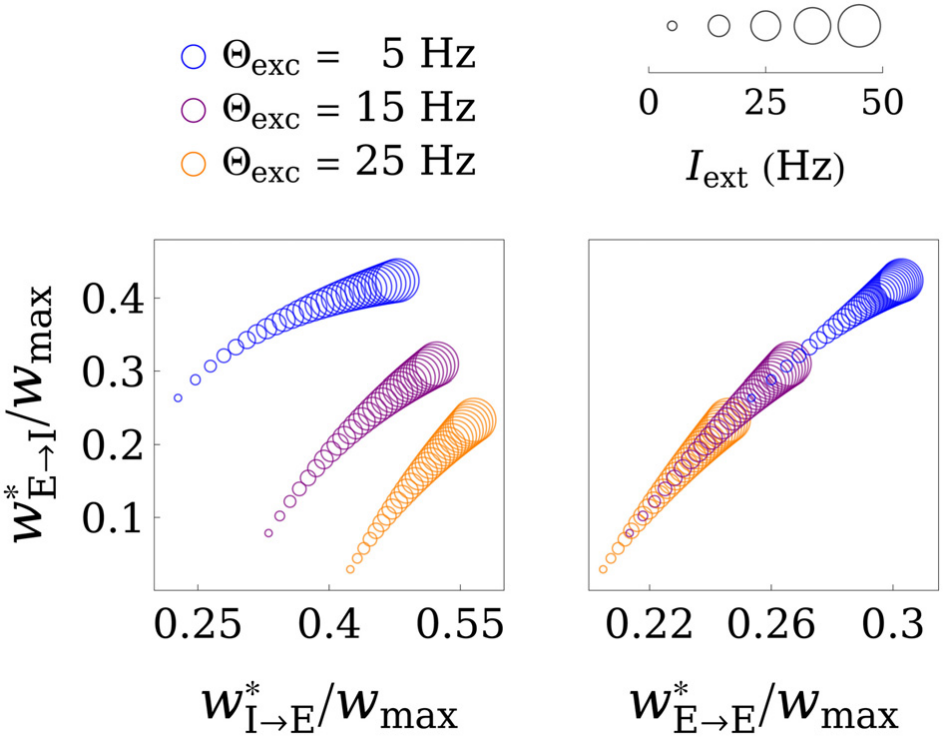
**Illustration of the fixed point in weight space**. The values of the final weights (in units of *w*_max_) are plotted as functions of the parameter Θ_exc_ and the training input strength *I*_ext_, where bigger circles correspond to greater *I*_ext_. The left panel shows the *w*_E→I_-*w*_I→E_ plane, while the right panel shows the *w*_E→I_-*w*_E→E_ plane. Interestingly, the fixed point in *w*_E→I_-*w*_E→E_ space only gets shifted along a line for different values of Θ_exc_ and *I*_ext_. For *I*_ext_ → ∞ the weights converge to a limit point, as is illustrated in Figure 4. For these plots, the parameters *A*_exc_ and Θ_inh_ were set to 2 and 18 Hz, respectively, and *w*_max_ was set to 4.

**Figure 4:**
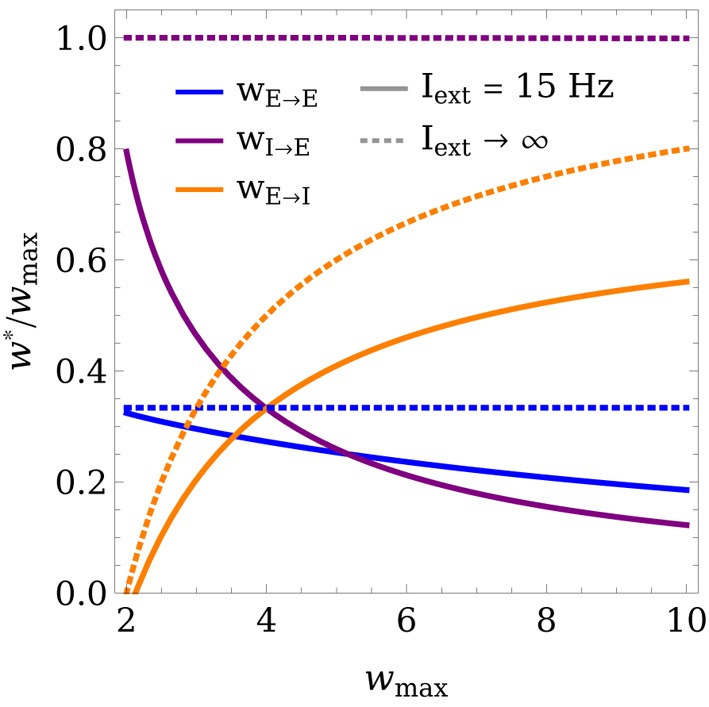
**Limit behavior of the fixed point of the weights for weak and strong inputs**. The final weight values (in units of *w*_max_) are plotted as a function of *w*_max_, both for *I*_ext_ = 15 Hz (solid lines) and in the limit of very large inputs *I*_ext_ → ∞ (dashed lines). In the limit case, *w*_E→E_ and *w*_I→E_ converge to expressions that are linear in *w*_max_, while *w*_E→I_ increases superlinearly. The learning rule parameters were set to Θ_exc_ = 6 Hz, Θ_inh_ = 18 Hz, and *A*_exc_ = 2.

Figure 3 shows the fixed points of the weight dynamics as a function of Θ_exc_, and the input strength *I*_ext_. Notably, w^*^_E→E_ and w^*^_E→I_ lie on a fixed line in the *w*_E→E_-*w*_E→I_ plane for all parameters Θ_*w*_ and *A*_*w*_. As the weight values are bounded by 0 and *w*_max_, the weights converge to a finite value for *I*_ext_ → ∞. This is also illustrated in Figure 4, which shows the final weight values as a function of *w*_max_, both for a finite training input and in the limit *I*_ext_ → ∞.

Importantly, the function of a WTA circuit critically depends on the strength of the recurrent connection *w*_E→E_ (Rutishauser et al., 2011). If *w*_E→E_ > 1, the network operates in “hard” mode, where only one unit can win at a time and the activation of all other units is zero. On the other hand, if *w*_E→E_ is smaller than 1, the network implements “soft” competition, which means that multiple units can be active at the same time. From Equation (27) (Methods) it follows that *w*_E→E_ > 1 is possible only if *w*_max_ > *A* + 1. As we will show in the following section, this condition is necessarily satisfied by learning rules that lead to stable WTA circuits.

#### 2.4.3. Parameter regimes for stable network function

We can now use the fixed points found in the previous section to express the condition for contraction given by condition (6) in terms of the learning rule parameters. In general, this new condition does not assume an analytically simple form. However, we can find simple sufficient conditions which still provide a good approximation to the actual value (see Methods section 4.2 for details). Specifically, as a key result of our analysis we derive the following sufficient condition: Convergence to a point in weight space that produces stable network dynamics is guaranteed if

(11) $$A_{\text{exc}}+b<w_{\text{max}}<2(1+A_{\text{exc}}),$$

where *b* is a parameter of the order 1, which is related to the minimum activation *x*_E_ (or the minimum non-zero input *I*_ext_) during training for which this condition should hold. If the minimum input *I*_min_ that the network will be trained on is known, then *b* can be computed from the fixed point x^*^_E,min_ = x^*^_E_(*I*_ext_ = *I*_min_), and set to *b* = Θ_exc_/x^*^_E,min_. This will guarantee contracting dynamics for the full range of training inputs *I*_ext_ ∈ [*I*_min_, ∞). In typical scenarios, *b* can be set to a number of the order 1. This is due to the fact that the network activation is roughly of the same order as the input strength. Setting Θ_exc_ to a value of similar order leads to *b* = Θ_exc_/x^*^_E,min_ ≈ 1.

**Figure 5:**
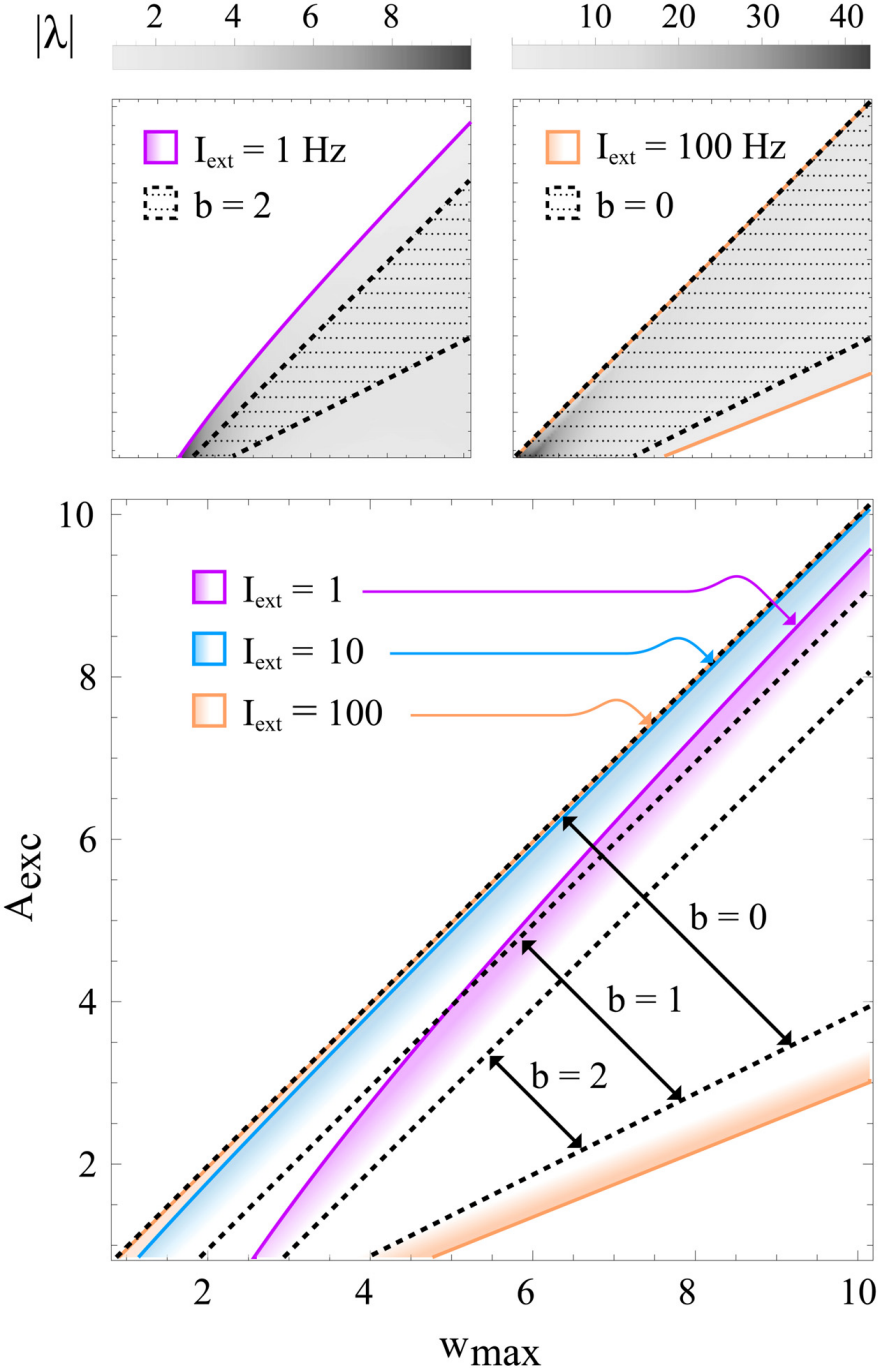
**Regions in learning rule parameter space that lead to a stable, contracting network**. All panels show the regions of stability in *w*_max_−*A*_exc_ space for different training input strengths. Colored lines correspond to exact solutions, while black, dotted lines correspond to the sufficient condition (11) for different values of *b*. The top panels illustrate that relatively small values of *b* (e.g., 2) roughly approximate the exact solution even for very small inputs (e.g., *I*_ext_ = 1 Hz; left), whereas *b* can be set to lower values (e.g., *b* = 0; right) if the input is larger. The gray-scale value represents the convergence rate |λ| (in units of *s*^−1^) of the activation dynamics for τ = 10 ms. The bottom panel shows in color the exact regions of contraction for inputs *I*_ext_ = 1, 10, 100 Hz and the approximation given by condition (11) for *b* = 0, 1, 2. Some of the colored regions (and dotted lines) correspond to the ones shown in the upper panels. It can be seen that for higher input strengths the upper bound on *A*_exc_ (or equivalently, the lower bound on *w*_max_) quickly converges to the *b* = 0 diagonal, which represents the asymptotic condition for *I*_exc_ → ∞. For these plots, the learning rule parameters were set to Θ_exc_ = 6 Hz and Θ_inh_ = 18 Hz.

Note that condition (11) is independent of Θ_exc_ and Θ_inh_. This is due to a simplification that is based on the assumption *A*_exc_ + *b* ≫ 1, which can be made without loss of generality. If *b* and *A*_exc_ are set to very low values, the full expressions given by 38 and (39) (see Methods section 4.2) apply instead. Figure 5 shows the the region defined by (11) for different *b* together with the exact condition for contraction, indicating that (11) is indeed sufficient and that *b* can safely be set to a value around 1 in most cases.

#### 2.4.4. Extension to multiple units

So far, we have only studied a small network that can be regarded as a single subunit of a larger, distributed WTA system. However, our results can be generalized to larger systems without much effort. In our model, as illustrated in Figures 1D,E, different localized WTA circuits can be coupled via excitatory projections. These projections include excitatory-to-inhibitory connections, as well as reciprocal connections between distant excitatory units. In order to demonstrate the effects of this coupling, we consider two localized subsystems, ***x*** = (*x*_E_, *x*_I_) and ***x***′ = (*x*′_E_, *x*′_I_), consisting of one excitatory and one inhibitory unit each. Furthermore, we add projections from *x*_E_ to *x*′_E_ and *x*′_I_, as required by our model. We denote by *w*_E→E′_ the strength of the long-range excitatory-to-excitatory connection, while we refer to the long-range excitatory-to-inhibitory connection as *w*_E→I′_. Note that for the sake of clarity we only consider the unidirectional case ***x*** → ***x***′ here, while the symmetric case ***x*** ↔ ***x***′ can be dealt with analogously.

We first look at the excitatory-to-inhibitory connections. If only *x*_E_ is active and *x*′_E_ is silent, then *x*_I_ and *x*′_I_ are driven by the same presynaptic population (*x*_E_), and *w*_E→I′_ converges to the same value as *w*_E→I_. Thus, after convergence, both inhibitory units are perfectly synchronized in their activation when *x*_E_ is active, and an equal amount of inhibition can be provided to *x*_E_ and *x*′_E_.

Besides synchronization of inhibition, proper WTA functionality also requires the recurrent excitation *w*_E→E′_ (between the excitatory populations of the different subunits) to converge to sufficiently low values, such that different units compete via the synchronized inhibition rather than exciting each other through the excitatory links. As pointed out by Rutishauser et al. (2012), the network is stable and functions correctly if the recurrent excitation between populations is lower than the recurrent self-excitation, i.e., *w*_E→E′_ < *w*_E′→E′_.

We now consider the case where *x*_E_ and *x*′_E_ receive an external input *I*_ext_. Whenever *x*′_E_ alone receives the input, there is no interaction between the two subunits, and the recurrent self-connection *w*_E′→E′_ converges to the value that was found for the simplified case of a single subunit (section 2.4.2). The same is true for the connection *w*_E→E_ if *x*_E_ alone receives the input. However, in this case *x*_E_ and *x*′_E_ might also interact via the connection *w*_E→E′_, which would then be subject to plasticity. As ***x*** projects to ***x***′, but not vice versa, we require *x*_E_ > *x*′_E_ if both *x*_E_ and *x*′_E_ receive the same input *I*_ext_, because *x*_E_ should suppress *x*′_E_ via the long-range competition mechanism. In terms of connection strengths, this means that w^*^_E→I′_ w^*^_I′→E′_ > w^*^_E→E′_, i.e., the inhibitory input to *x*′_E_ that is due to *x*_E_ must be greater than the excitatory input *x*′_E_ receives from *x*_E_. In the Methods (section 4.3), we show that a sufficient condition for this to be the case is

(12) $$w_{\text{max}}>A+b+1,$$

which alters our results from section 2.4.3 only slightly, effectively shifting the lower bound on *w*_max_ by an offset of 1, as can be seen by comparing conditions (11) and (12). On the other hand, making use of the fact that *x*′_E_ < *x*_E_, it can be shown that *w*_E→E′_ converges to a value smaller than *w*_E′→E′_ (see Methods section 4.3), as required by the stability condition mentioned above.

### 2.5. Gain control and normalization

In the previous section, we showed how synaptic plasticity can be used to drive the connection strengths toward regimes which guarantee stable network dynamics. Since the actual fixed point values of the weights change with the training input, this mechanism can as well be used to tune certain functional properties of the network. Here we focus on controlling the gain of the network, i.e., the relationship between the strength of the strongest input and the activation of the winning excitatory units within the recurrent circuit, as a function of the training input.

In the case of a single active population, the gain is given by Λ = *x*_E_/*I*_ext_ = (1 − w^*^_E→E_ + w^*^_E→I_ w^*^_I→E_)^−1^, as can be inferred from Equation (7). Depending on the gain, the network can either amplify (Λ > 1) or weaken (Λ < 1) the input signal.

**Figure 6:**
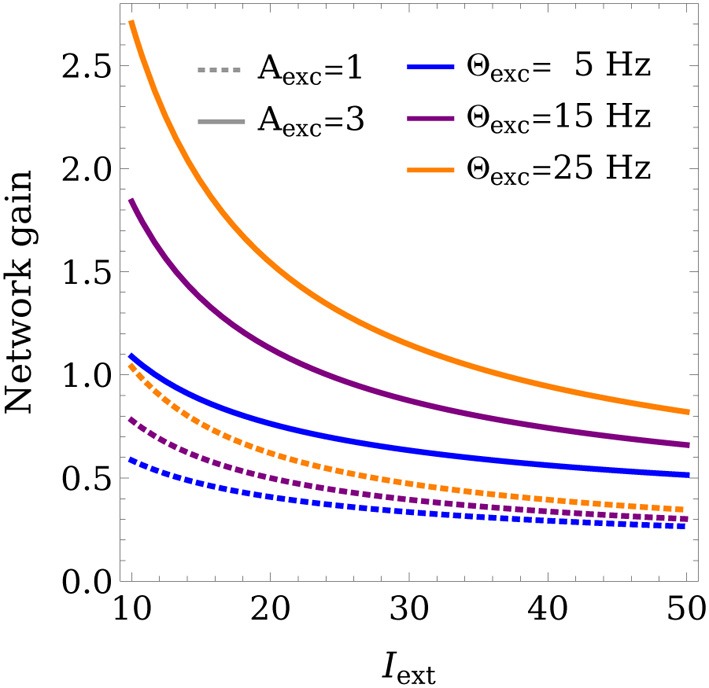
**Control of the network gain**. The network gain is plotted for different learning rule parameters Θ_exc_, *A*_exc_ as a function of the training input strength *I*_ext_. Θ_inh_ was set to 18 Hz and *w*_max_ was set to 4.

Figure 6 shows how the gain varies as a function of the learning rule parameters and the training input strength *I*_ext_. Low average input strengths cause the weights to converge to values that lead to an increased gain, while higher training inputs lower the gain. This can be regarded as a homeostatic mechanism, acting to keep the network output within a preferred range. This provides a mechanism for the network to adapt to a wide range of input strengths, while still allowing stable WTA competition.

### 2.6. Simulation results

As a final step, we verify the analytical results in software simulations of a distributed, plastic WTA network, as illustrated in Figures 1D,E. Note that here we consider the case where two subgroups are coupled bidirectionally via excitatory long-range projections, while in section 2.4.4, for the sake of clarity, we focus on the unidirectional case. The desired functionality of the resulting network is global competition between the excitatory populations, i.e., the population that receives the strongest input should suppress activation of the other populations, even if the excitatory populations are not directly competing via the same, local inhibitory population. We consider a network with two groups, each consisting of two excitatory populations and one inhibitory population (see Figure 1E). While the excitatory populations are connected in an all-to-all manner, inhibitory populations can only target the excitatory populations within their local groups, but do not form long-range projection. Initially, all connection weights (excitatory and inhibitory) are set to random values between 0.3 and 1.8. Note that those values could potentially violate the conditions for contraction defined in (6), but we will show empirically that the plasticity mechanism can still drive the weights toward stable regimes. As training input, we present 1000 constant patterns for 2 s each. In every step, four input values in the ranges 5 ± 2 Hz, 10 ± 2 Hz, 15 ± 2 Hz, and 20 ± 2 Hz are drawn from uniform distributions and applied to the four excitatory units. The different input signals are randomly assigned to the populations in every step, such that a randomly chosen population receives the strongest input. Thereby, each population only receives one of the four inputs.

**Figure 7:**
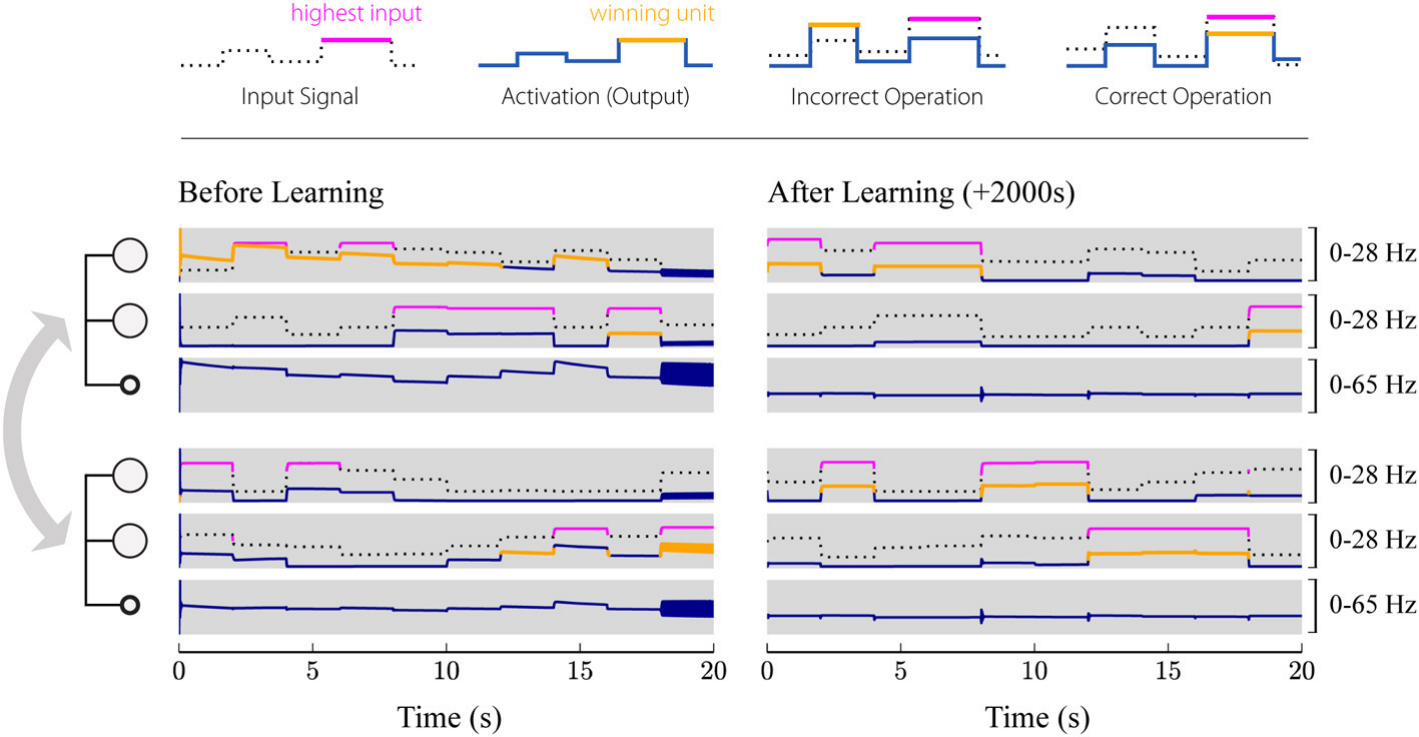
**Simulated evolution of the plastic network**. 1000 input patterns were applied for 2 s each. Two local subsystems, consisting of two excitatory units and one inhibitory unit each, are coupled via all-to-all excitatory connections, while inhibitory feedback is provided only locally. The first three rows show the populations of the first group, where the first two rows correspond to the two excitatory populations, and the third row shows the activation of the inhibitory population. The last three rows show the activations of the second group. The left panel shows the first 20 s after initialization (before learning), while the right panel shows the network activity during the last 20 s (after learning). Solid blue and orange lines correspond to the firing rate of the respective population, whereby the highlighted segments (orange) mark the winner among the four excitatory populations. The input given to the individual units is plotted as a dotted black or solid magenta line, where the highlighted segments (magenta) correspond to the strongest signal among the four. Thus, the network operates correctly if magenta and orange lines are aligned (the one population that receives the strongest input wins the competition), while misaligned lines (the population receiving the strongest input does not win the competition) indicate incorrect operation. Initially, the network frequently selects the wrong winning unit and even starts oscillating for some input patterns (around 18 s). After training (right), the network converges to a stable state with only the winning unit active for different input patterns.

Figure 7 shows the activation of the different populations before and after learning. Before learning (left), the network does not necessarily implement stable competition between the different excitatory populations. Instead, it may end up in an oscillating state or amplify the wrong winning unit. However, after training (Figure 7, right), the network always converges to a stable state representing the winner of the competition. Furthermore, it can be seen that the inhibitory populations perfectly synchronize, as described in section 2.4.4.

**Figure 8:**
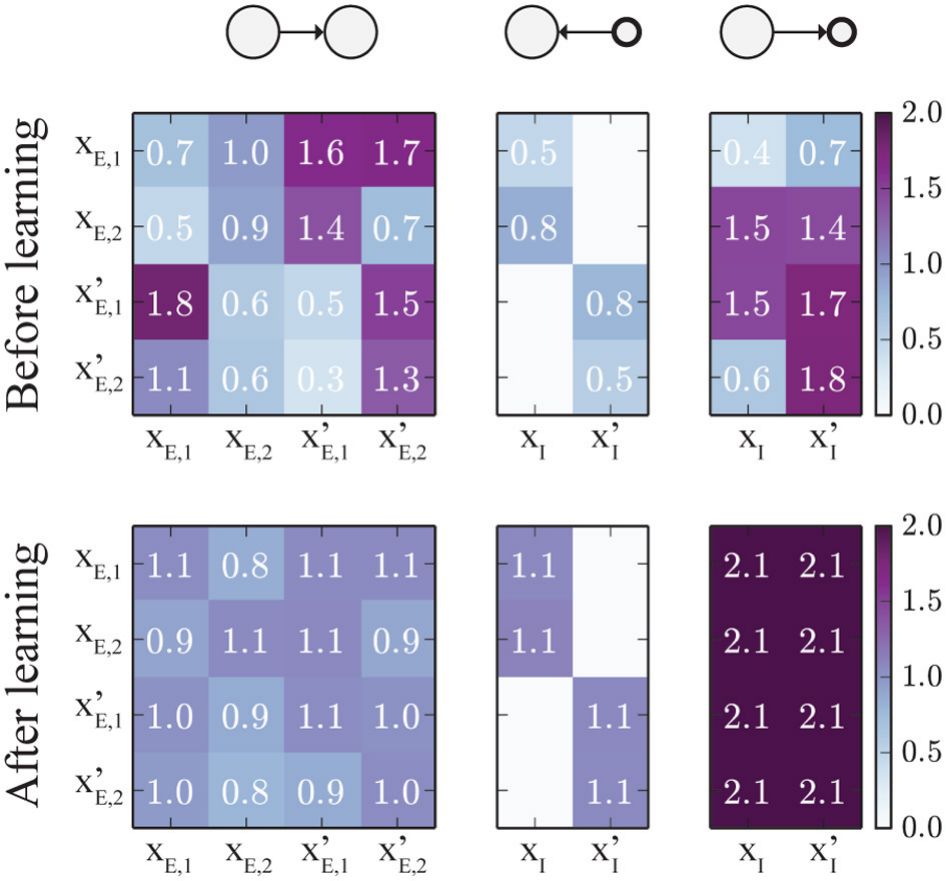
**Evolution of the connectivity matrices**. The top panels show the (initially random) weight matrices, and the lower panels show the final weight matrices after 2000 s of simulated training time. The left panel shows the connections between excitatory populations. The panel in the middle represents the connections from inhibitory to excitatory populations. Note that some inhibitory connections are set to zero as inhibitory units can only project to targets within their own local group, according to our model. The right panel shows the connections from excitatory to inhibitory units. Here, connections from the same source converge to the same value, leading to perfect synchronization of the two inhibitory units. The (rounded) weight values are displayed on top of the image.

The change of weights is illustrated in Figure 8: Initially (top), all weights were set to random values in the range [0.3, 1.8]. Since all populations receive the same average input, the weight matrices should converge to symmetric states. For the specific set of learning rule parameters we chose in this example, and the specific input rates described above, *w*_E→E_ converges to a value around 1, which means that the network is at the edge of the transition between hard and soft WTA behavior. The weights *w*_E→I_, connecting excitatory to inhibitory units, converge to values around 2. Furthermore, the weights *w*_I→E_, which connect inhibitory to excitatory units all converge to very similar values (around 1.1), such that inhibition is synchronized across the whole network. Note that not all connections between excitatory populations have converged to the same value. This is because as soon as the network is close to the hard WTA regime, some connections cannot change anymore as only one excitatory unit is active at a time, and the weight change is zero if either the pre- or the post-synaptic unit is inactive.

## 3. Discussion

We have shown how neural circuits of excitatory and inhibitory neurons can self-organize to implement stable WTA competition. This is achieved through an interplay of excitatory and inhibitory plasticity mechanisms operating on all synapses of the network. As a key result, we provide analytical constraints on the learning rule parameters, which guarantee emergence of the desired network function.

Although constraints on the weights for stable competition in recurrent excitatory-inhibitory circuits have been derived before (Xie et al., 2002; Hahnloser et al., 2003; Rutishauser and Douglas, 2009; Rutishauser et al., 2012), it has remained unclear how a network can self-tune its synaptic weights to comply with these conditions. The presented model achieves this and provides important insights regarding the mechanisms responsible for this self-tuning. Our results predict a relationship between the maximum synaptic weight *w*_max_ in a circuit and the learning rule parameter *A*_exc_, which controls the contribution of the presynaptic rate to the shifting of the threshold between potentiation and depression. Furthermore, our model predicts a relationship between the network gain and the amount of excitatory input into the circuit during development or training (see Figure 6), indicating that high gain (amplification) should be expected for weak inputs, and low gain for strong inputs, which is in accordance with common assumptions about homeostasic mechanisms (Turrigiano, 2011).

From a developmental perspective, the self-configuration of functional WTA circuits through plasticity has the advantage of requiring a smaller number of parameters to be encoded genetically to obtain stable and functional network structures (Zubler et al., 2013). With self-tuning mechanisms like the ones suggested here, only the parameters for the two different types of plasticity in excitatory and inhibitory synapses, rather than the strengths of all synaptic connections, need to be specified, and the network can adapt to the statistics of inputs it receives from its environment and from other brain regions.

Besides guaranteeing stability, it is also desirable to control functional properties of the circuit, such as its gain. Experimental data suggests that cortical recurrent circuits often operate in a high gain regime and with strong (larger than unity) recurrent excitatory feedback (Douglas et al., 1995). The strength of this feedback determines whether the WTA is “soft” (multiple excitatory units can be active at the same time) or “hard” (only one unit can be active at a time, i.e., the network operates in a nonlinear regime) (Rutishauser et al., 2011). Many interesting computations that can be realized with these types of networks rely on the non-linearities introduced by such strong recurrent excitation (e.g., Vapnik, 2000), therefore it is important that similar conditions can be achieved with our model. In addition, various forms of learning rely on balanced WTA competition (Masquelier et al., 2009; Habenschuss et al., 2012; Nessler et al., 2013), which requires an adaptation of the gain as the excitatory connections into the circuit undergo plasticity. In our network, the resulting network gain is a function of both the learning rule parameters and the strength of the training input signals. As a consequence, our system can switch between high and low gain, and hard or soft WTA behavior simply by receiving input stimuli of different (average) strengths. Thus, different parts of the network might develop into different functional modules, depending on the inputs they receive.

Our model does not specifically address the question of how the network structure, which leads to our results (essentially random all-to-all connectivity) might develop in the first place. For instance, if certain long-range connections between multiple subcircuits do not exist initially, they will never be established by our model, and the units of the different subcircuits can never compete. On the one hand, this might be a desired effect, e.g., to construct hierarchies or asymmetric structures for competition, in which some parts of the network are able to suppress other parts, but not vice-versa. On the other hand, structural plasticity could account for the creation of missing synaptic connections, or the removal of ineffective connections if the desired stable function cannot be achieved with the anatomical substrate. There is increasing evidence for activity dependent synapse formation and elimination in both juvenile and adult brains (Butz et al., 2009), in particular a coordinated restructuring of inhibitory and excitatory synapses for functional reorganization (Chen and Nedivi, 2013). Another approach, recently investigated in simulations by Bauer (2013), is to set up the right network topology by developmental self-construction processes in a first step, and the tune the network using synaptic plasticity in a second step.

Our model is based on a weight-dependent variation of the learning rule proposed by Pfister and Gerstner (2006), but this is by no means the only learning rule capable of the self-calibration effect we describe in this article. By changing its parametrization, the rule can subsume a wide variety of commonly used Hebbian, STDP-like, and homeostatic plasticity mechanisms. Indeed, further experiments, which are not presented in this manuscript, indicate that a whole class of learning rules with depression at low and potentiation at high postsynaptic firing rates would lead to similar results. We chose the triplet rule to demonstrate our findings as its parameters have been mapped to experiments, and also because it can be written in an analytically tractable form. We have assumed here a specific type of inhibitory plasticity, which analytically is of the same form as the simultaneous excitatory plasticity, but uses different parameters. With the parameters we chose for the inhibitory plasticity rule, we obtain a form that is very similar to the one proposed by Vogels et al. (2011). By introducing inhibitory plasticity it is no longer necessary to make common but biologically unrealistic assumptions, like pre-specified constant and uniform inhibitory connection strengths (Oster et al., 2009), or more abstract forms of summing up the excitatory activity in the circuit (Jug et al., 2012; Nessler et al., 2013), because inhibitory weights will automatically converge toward stable regions. Inhibitory plasticity has received more attention recently with the introduction of new measurement techniques, and has revealed a great diversity of plasticity mechanisms, in line with the diversity of inhibitory cell types (Kullmann and Lamsa, 2011; Kullmann et al., 2012). Our model involves only a single inhibitory population per local sub-circuit, which interacts with all local excitatory units. Not only is this a common assumption in most previous models, and greatly simplifies the analysis, but also is in accordance with anatomical and electrophysiological results of relatively unspecific inhibitory activity in sensory cortical areas (Kerlin et al., 2010; Bock et al., 2011). However, recent studies have shown more complex interactions of different inhibitory cell types (Pfeffer et al., 2013), making models based on diverse cell types with different properties an intriguing target for future studies. The assumption of a common inhibitory pool that connects to all excitatory units is justified for local circuits, but violates anatomical constraints on the length of inhibitory axons if interacting populations are far apart (Binzegger et al., 2005). Our results easily generalize to the case of distributed inhibition, by adapting the model of Rutishauser et al. (2012) (see Figure 1E). Our contribution is to provide the first learning theory for these types of circuits.

Since our model is purely rate-based, a logical next step is to investigate how it translates into the spiking neural network domain. Establishing similar constraints on spike-based learning rules that enable stable WTA competition remains an open problem for future research, although Chen et al. (2013) have shown empirically that WTA behavior in a circuit with topologically ordered input is possible under certain restrictions on initial synapse strengths, and in the presence of STDP and short-term plasticity. Spiking WTA circuits can potentially utilize the richer temporal dynamics of spike trains in the sense that the order of spikes and spike-spike correlations have an effect on the connectivity.

Potential practical applications of our model, and future spiking extensions, lie in neuromorphic VLSI circuits, which have to deal with the problem of device mismatch (Indiveri et al., 2011), and can thus not be precisely configured a priori. Our model could provide a means for the circuits to self-tune and autonomously adapt to the peculiarities of the hardware.

## 4. Materials and methods

### 4.1. Derivation of the plasticity mechanism

The learning rule given by Equation (3) is based on the triplet STDP rule by Pfister and Gerstner (2006). Since we are interested in the rate dynamics, we use the mean-field approximation of this rule, which is provided by the authors and leads to an expected weight change of

(13) $$\begin{array}{l} \dot{w}=x_{\text{pre}}x_{\text{post}}(A_{2}^{+}\tau_{+}-A_{2}^{-}\tau_{-}+A_{3}^{+}\tau_{+}\tau_{y}x_{\text{post}}\\ \;-A_{3}^{-}\tau_{-}\tau_{x}x_{\text{pre}}), \end{array}$$

where *x*_pre_, *x*_post_ are the pre- and postsynaptic activations and *A*^±^_2_, *A*^±^_3_, τ_±_, τ_*x, y*_ are parameters that determine the amplitude of weight changes in the triplet STDP model. All of the parameters are assumed to be positive. Through a substitution of constants given by

(14) $$\tau_{\text{s}}^{2}:=A_{3}^{+}\tau_{+}\tau_{y},$$

(15) $$\Theta_{w}:=\left(A_{2}^{-}\tau_{-}-A_{2}^{+}\tau_{+}\right)/\tau_{\text{s}}^{2},$$

(16) $$A_{w}:=A_{3}^{-}\tau_{-}\tau_{x}/\tau_{\text{s}}^{2},$$

the rule in Equation (13) can be written in the simpler form

(17) $$\dot{w}=\tau_{\text{s}}^{2}x_{\text{pre}}x_{\text{post}}\left(x_{\text{post}}-(\Theta_{w}+A_{w}x_{\text{pre}})\right),$$

where Θ_*w*_ is in units of a firing rate and *A*_*w*_ is a unitless constant. The terms in parentheses on the right of Equation (17) can be divided into a positive (LTP) part that depends on *x*_post_, and a negative (LTD) part that depends on *x*_pre_. In order to constrain the range of weights, we add weight-dependent terms *m*_+_(*w*) and *m*_−_(*w*) to the two parts of the rule, which yields

(18) $$\dot{w}=\tau_{\text{s}}^{2}x_{\text{pre}}x_{\text{post}}\left(x_{\text{post}}m_{+}(w)-(\Theta_{w}+A_{w}x_{\text{pre}})m_{-}(w)\right).$$

Throughout this manuscript, we use a simple, linear weight dependence *m*_+_ = *w*_max_ − *w* and *m*_−_ = *w*, which effectively limits the possible values of weights to the interval [0, *w*_max_]. We chose this form, which is described by a single parameter, for reasons of analytical tractability and because it is consistent with experimental findings (Gütig et al., 2003). In Pfister and Gerstner (2006), values for the parameters τ_*x, y*_, τ_±_, and *A*^±^_2,3_ of the rule Equation (13) were determined from fits to experimental measurements in pyramidal cells in visual cortex (see Table 1) and hippocampal cultures (Bi and Poo, 1998, 2001; Sjöström et al., 2001; Wang et al., 2005). We used these values to calculate plausible values for Θ_*w*_, *A*_*w*_, and τ_*s*_ using Equations (14) to (16). In our simulations, we use parameters very similar to the experimentally derived values in Table 1. Specifically, for inhibitory connections we use parameters very similar to the ones found from fits of experimental data to the triplet STDP model with all-to-all spike interactions. On the other hand, we choose parameters for the excitatory plasticity rules which are close to fits of the triplet STDP rule with nearest-neighbor spike interactions. The parameters that were used in software simulations and to obtain most of the numeric results are listed in Table 2. Note that for the weight-dependent rule in Equation (18) we have assumed that the parameter Θ_*w*_ influences only the LTD part. According to the definition in Equation (15), this is the case if *A*^−^_2_ ≫ *A*^+^_2_, or Θ_*w*_ ≈ *A*^−^_2_ τ_−_/τ_*s*_, respectively. Otherwise Θ_*w*_ contains both a potentiating (*A*^+^_2_) and a depressing (*A*^−^_2_) component, and Equation (18) should be replaced with a more complex expression of the form of Equation (13).

**Table 2:** **Model parameters used in software simulation**.

**Parameter**	**Value**	**Description**
Θ_exc_	6 Hz	Learning rule parameter
Θ_inh_	18 Hz	Learning rule parameter
*A*_exc_	2	Learning rule parameter
*w*_max_	4	Maximum weight value
τ^2^_*s*,exc_	3.6 ms^2^	Exc. connection learning rate parameter
τ^2^_*s*,inh_	1.3 ms^2^	Inh. connection learning rate parameter
τ_exc_	5 ms	Exc. population time constant
τ_inh_	1 ms	Inh. population time constant

### 4.2. Derivation of the stability criteria

In section 2.4, we outlined how the fixed points and stability criteria for the WTA system can be found. In this section, we provide the detailed derivations that led to these results.

As described in section 2.4, we first consider a simplified system of one excitatory and one inhibitory population, *x*_E_ and *x*_I_, which yield an activation vector ***x*** = (*x*_E_, *x*_I_)_*T*_. They are coupled recurrently through a weight matrix $W=\left[\begin{array}{cc} w_{\text{E }\to \text{ E}} & w_{\text{I }\to \text{ E}}\\ w_{\text{E }\to \text{ I}} & 0 \end{array}\right]$, receive external inputs *I*_ext_(*t*) with weights μ_E_ and μ_I_ respectively, and have thresholds *T*_E_, *T*_I_. Assuming that both units are active, i.e., their total synaptic input is larger than their thresholds, their dynamics are described by

(19) $$\tau_{\text{exc}}{\dot{x}}_{\text{E}}=-x_{\text{E}}+w_{\text{E}\to \text{E}}x_{\text{E}}-w_{\text{I}\to \text{E}}\;x_{\text{I}}+\mu_{\text{E}}I_{\text{ext}}-T_{\text{E}},$$

(20) $$\tau_{\text{inh}}{\dot{x}}_{\text{I}}=-x_{\text{I}}+w_{\text{E}\to \text{I}}x_{\text{E}}+\mu_{\text{I}}I_{\text{ext}}-T_{\text{I}},$$

where τ_exc_, τ_inh_ are the population time constants. The fixed points of the activations can be found by setting *ẋ*_E_ = *ẋ*_I_ = 0. If we assume, for simplicity, that *T*_E_ = *T*_I_ = 0 this yields the fixed points

(21) $$x_{\text{E}}^{*}=\Lambda I_{\text{ext}}\left(\mu_{\text{E}}-w_{\text{I}\to \text{E}}\mu_{\text{I}}\right),$$

(22) $$x_{\text{I}}^{*}=\Lambda I_{\text{ext}}\left(w_{\text{E}\to \text{I}}\mu_{\text{E}}-(w_{\text{E}\to \text{E}}-1)\mu_{\text{I}}\right).$$

where

(23) $$\Lambda ={\left(1-w_{\text{E}\to \text{E}}+w_{\text{E}\to \text{I}}w_{\text{I}\to \text{E}}\right)}^{-1}$$

is the network gain. Furthermore, we can make the assumption that μ_I_ = 0 and μ_E_ = 1, effectively disabling the external input to the inhibitory population. This reduces Equations (21) and (22) to

(24) $$x_{\text{E}}^{*}=\Lambda I_{\text{ext}},$$

(25) x=I∗ΛwE→IIext.

These simplifications do not change the results of our analysis qualitatively and can be made without loss of generality.

Approximating *x*_pre_ and *x*_post_ by their fixed point activities (as described in section 2.4), and setting *ẇ* = 0 in the learning rule Equation (18), the fixed point of the weight dynamics (with *w* > 0) takes the form

(26) $$w^{*}=\frac{w_{\text{max}}x_{\text{post}}^{*}}{\Theta_{w}+A_{w}x_{\text{pre}}^{*}+x_{\text{post}}^{*}}.$$

Note that this fixed point in weight space always exists for any given *x*_pre_ and *x*_post_, and is stable for the weight dependence *m*_+_(*w*) = *w*_max_ − *w*; *m*_−_(*w*) = *w* that we chose in Equation (18). In fact, this is true for all choices of the weight dependence satisfying ∂*m*_+_/∂*w* < 0 and ∂*m*_−_/∂*w* > 0, as can be shown by means of a linear stability analysis.

We now derive the fixed points for the weights *w*_E→E_, *w*_E→I_, and *w*_I→E_ of the simplified system. For *w*_E→E_, Equation (26) can be simplified by noting that x^*^_pre_ = x^*^_post_ = x^*^_E_, leading to an expression that depends on the activation of the excitatory population x^*^_E_:

(27) $$w_{\text{E }\to \text{ E}}^{*}=\frac{w_{\text{max}}}{\Theta_{\text{exc}}/x_{\text{E}}^{*}+A_{\text{exc}}+1}.$$

Similarly, we can compute the fixed point of *w*_E→I_ as a function of x^*^_E_, noting that x^*^_post_ = x^*^_I_ = *w*_E→I_x^*^_E_ [see Equations (24) and (25)]:

(28) $$w_{\text{E }\to \text{ I}}^{*}=w_{\text{max}}-\Theta_{\text{exc}}/x^{*}-A_{\text{exc}}.$$

Finally, using the relationship x^*^_I_ = *w*_E→I_x^*^_E_ from Equations (24) and (25), and the previously computed value of *w*_E→I_ from Equation (28) with the fixed point equation for *w*_I→E_, we obtain

(29) $$w_{\text{I }\to \text{ E}}^{*}=\frac{w_{\text{max}}}{\Theta_{\text{inh}}/x_{\text{E}}^{*}-A_{\text{inh}}\left(\Theta_{\text{exc}}/x_{\text{E}}^{*}+(A_{\text{exc}}-w_{\text{max}})\right)+1}.$$

In the following, we set *A*_inh_ = 0, as described in section 2.3. An exact solution for the activation x^*^_E_ at the fixed point of the system is obtained by inserting w^*^_E→E_, w^*^_E→I_, and w^*^_I→E_ into Equation (24), and solving the resulting fixed-point problem x^*^_E_ = *f*(x^*^_E_). This corresponds to finding the roots of the third order polynomial

(30) $$P(x)=a_{0}+a_{1}x+a_{2}x^{2}+a_{3}x^{3}=0$$

with coefficients

(31) $$a_{0}=\Theta_{\text{exc}}\Theta_{\text{inh}}I_{\text{ext}},$$

(32) $$\begin{array}{l} a_{1}=-\Theta_{\text{exc}}\Theta_{\text{inh}}+\Theta_{\text{exc}}I_{\text{ext}}+\Theta_{\text{inh}}I_{\text{ext}}+\Theta_{\text{inh}}A_{\text{exc}}I_{\text{ext}}\\ \;+\Theta_{\text{exc}}^{2}w_{\text{max}}, \end{array}$$

(33) $$\begin{array}{l} a_{2}=-\Theta_{\text{exc}}-\Theta_{\text{inh}}-\Theta_{\text{inh}}A_{\text{exc}}+I_{\text{ext}}+A_{\text{exc}}I_{\text{ext}}+\Theta_{\text{exc}}w_{\text{max}}\\ \;+\Theta_{\text{inh}}w_{\text{max}}+2\Theta_{\text{exc}}A_{\text{exc}}w_{\text{max}}-\Theta_{\text{exc}}w_{\text{max}}^{2}, \end{array}$$

(34) $$\begin{array}{l} a_{3}=-1-A_{\text{exc}}+w_{\text{max}}+A_{\text{exc}}w_{\text{max}}+A_{\text{exc}}^{2}w_{\text{max}}-w_{\text{max}}^{2}\\ \;-A_{\text{exc}}w_{\text{max}}^{2}. \end{array}$$

The activation of the excitatory population *x*_E_ at the fixed point is then given by the positive, real root of Equation (30).

The fixed point of the activation x^*^_E_, and thus the fixed points of the weights, are monotonic functions of the training input strength *I*_ext_ (see Figure 3, for example). In the following, we investigate the behavior of the fixed point weight values for very large and very small external inputs during training, respectively. This helps us to find conditions on the learning rule parameters that lead to stable dynamics (of the network activation) for any training input strength. We define a positive constant *b* := Θ_exc_/x^*^_E_, and plug it into Equations (27)–(29). This yields

(35) $$w_{\text{E }\to \text{ E}}^{*}=\frac{w_{\text{max}}}{A_{\text{exc}}+b+1},$$

(36) $$w_{\text{E }\to \text{ I}}^{*}=w_{\text{max}}-A_{\text{exc}}-b,$$

(37) $$w_{\text{I }\to \text{ E}}^{*}=\frac{w_{\text{max}}\Theta_{\text{exc}}}{b\Theta_{\text{inh}}+\Theta_{\text{exc}}}.$$

Inserting Equations (35)–(37) into the condition for contraction of the activation dynamics given by (6), we can describe the condition in terms of the learning rule parameters, and a new constant $\tilde{\Theta }:=\Theta_{\text{exc}}/(\Theta_{\text{exc}}+b\Theta_{\text{inh}})$:

(38) $$\;\frac{1}{\left(1+A_{\text{exc}}+b\right)}\;<\tilde{\Theta }<1,$$

(39) $$\begin{array}{l} (A_{\text{exc}}+b)\left(1+\frac{1}{\tilde{\Theta }{\left(1+A_{\text{exc}}+b\right)}^{2}-1}\right)<w_{\text{max}}\\ \;<2(1+A_{\text{exc}}+b), \end{array}$$

Assuming *A*_exc_ + *b* ≫ 1 (note that we can always set *A*_exc_ to a sufficiently large value), the conditions reduce to

(40) $$0<\tilde{\Theta }<1,$$

(41) $$A_{\text{exc}}+b<w_{\text{max}}<2(1+A_{\text{exc}}+b),$$

whereby the first condition can be dropped, since $\tilde{\Theta }\in [0,1]$ always holds. The second condition still depends on *b*, and therefore on x^*^_E_. We will illustrate how to eliminate this dependence under very weak assumptions. First, in the limit of very large inputs x^*^_E_ also takes very large values, leading to *b* → 0 for *I*_ext_ → ∞. In that case, condition (41) becomes independent of *b* and can be written as

(42) $$A_{\text{exc}}<w_{\text{max}}<2(1+A_{\text{exc}}).$$

On the other hand, in the case of very small inputs we have to include the effects of *b*, as *b* can in principle take very large values. In typical scenarios the output of the network can be assumed to be roughly of the order of its input. If Θ_exc_ is chosen to be of the same order, then *b* ≈ 1. For any finite *b*, we can express the stability condition that is valid for all inputs as the intersection of the conditions for large inputs, condition (42), with the one for arbitrarily small inputs, condition (41), leading to

(43) $$A_{\text{exc}}+b<w_{\text{max}}<2(1+A_{\text{exc}}).$$

Note that this condition can be met for any finite *b* by choosing sufficiently large *A*_exc_ and *w*_max_. However, as discussed above, choices of the parameter *b* of the order 1 should be sufficient for typical scenarios, whereas higher values would guarantee stable dynamics for very low input strengths (e.g., *I*_ext_ ≪ Θ_exc_). This is illustrated in Figure 5, where the exact regions of stability as a function of *w*_max_ and *A*_exc_ are shown for different training input strengths, together with the sufficient conditions given by (43). In practice, a good starting point for picking a value *b* for which the stability conditions should hold is to determine the minimum non-zero input *I*_min_ encountered during training for which this condition should hold, and setting *b* = Θ_exc_/x^*^_E,min_, where x^*^_E,min_ is the fixed point activation for *I*_ext_ = *I*_min_.

### 4.3. Extension to multiple units

In this section, we illustrate how multiple subunits, as analyzed in the previous section, can be combined to larger WTA networks with distributed inhibition. For the sake of simplicity, we only consider the unidirectional case, where a subunit ***x*** = (*x*_E_, *x*_I_) projects onto another subunit ***x***′ = (*x*′_E_, *x*′_I_) via excitatory connections *w*_E→E′_ and *w*_E→I′_. The bidirectional case ***x*** ↔ ***x***′ can be analyzed analogously. If *x*_E_ and *x*′_E_ receive the same input, the response of *x*′_E_ should be weaker, such that activation of *x*_E_ causes suppression of *x*′_E_ rather than excitation. This means that

(44) $$w_{\text{E}\to {\text{E}}^{\prime }}^{*}<w_{\text{E}\to {\text{I}}^{\prime }}^{*}w_{{\text{I}}^{\prime }\to {\text{E}}^{\prime }}^{*}$$

must hold. We assume that both subsystems have been trained on inputs of the same average strength, such that their local connections have converged to the same weights, i.e., w^*^_E′→I′_ = w^*^_E→I_ and w^*^_I′→E′_ = w^*^_I→E_. Furthermore, we assume that condition (44) is true initially. This can be guaranteed by setting the initial value of *w*_E→E′_ to a sufficiently small number. Our task then is to show that condition (44) remains true for all time. The values of w^*^_E′→I′_ and w^*^_I′→E′_, or w^*^_E→I_ and w^*^_I→E_ respectively, are described by Equations (36) and (37). On the other hand, according to Equation (26), the value of w^*^_E→E′_ is given by

(45) $$w_{\text{E}\to {\text{E}}^{\prime }}^{*}=\frac{w_{\text{max}}x_{\text{E}}^{'*}}{\Theta_{\text{exc}}+A_{\text{exc}}x_{\text{E}}^{*}+x_{\text{E}}^{'*}}.$$

Plugging all this into condition (44) and simplifying the expression, leads to the condition

(46) $$w_{\text{max}}>A_{\text{exc}}+b+\frac{{x^{\prime }}_{\text{E}}}{A_{\text{exc}}x_{\text{E}}+{x^{\prime }}_{\text{E}}+\Theta },$$

which can be replaced by the sufficient condition

(47) $$w_{\text{max}}>A_{\text{exc}}+b+1,$$

that guarantees x^*^_E′_ < x^*^_E_ if both excitatory populations receive the same input. On the other hand, this result implies w^*^_E→E′_ < w^*^_E′→E′_, which is required for stable network dynamics (Rutishauser et al., 2012), and can be verified by comparing the respective fixed point equations

(48) $$w_{\text{E}\to {\text{E}}^{\prime }}^{*}={x^{\prime }}_{\text{E}}/\left(\Theta_{\text{exc}}+A_{\text{exc}}x_{\text{E}}+{x^{\prime }}_{\text{E}}\right),$$

(49) $$w_{{\text{E}}^{\prime }\to {\text{E}}^{\prime }}^{*}={x^{\prime }}_{\text{E}}/\left(\Theta_{\text{exc}}+A_{\text{exc}}{x^{\prime }}_{\text{E}}+{x^{\prime }}_{\text{E}}\right).$$

### 4.4. Software simulation

Software simulations of our model were implemented using custom Python code based on the “NumPy” and “Dana” packages, and run on a Linux workstation. Numerical integration of the system dynamics was carried out using the forward Euler method with a 1 ms timestep.

## Author contributions

Jonathan Binas, Ueli Rutishauser, Giacomo Indiveri, Michael Pfeiffer conceived and designed the experiments. Jonathan Binas performed the experiments and analysis. Jonathan Binas, Ueli Rutishauser, Giacomo Indiveri, Michael Pfeiffer wrote the paper.

## Funding

The research was supported by the Swiss National Science Foundation Grant 200021_146608, and the European Union ERC Grant “neuroP” (257219).

### Conflict of interest statement

The authors declare that the research was conducted in the absence of any commercial or financial relationships that could be construed as a potential conflict of interest.
